# Discovery of Pinostrobin as a Melanogenic Agent in cAMP/PKA and p38 MAPK Signaling Pathway

**DOI:** 10.3390/nu14183713

**Published:** 2022-09-09

**Authors:** Jeong-Hyun Yoon, Kumju Youn, Mira Jun

**Affiliations:** 1 Department of Health Sciences, The Graduate School of Dong-A University, Busan 49315, Korea; 2 Department of Food Science and Nutrition, Dong-A University, Busan 49315, Korea; 3 Center for Food & Bio Innovation, Dong-A University, Busan 49315, Korea

**Keywords:** melanogenesis, pinostrobin, MITF, melanogenesis-related enzyme, cAMP/PKA, p38

## Abstract

Melanogenesis is the process of melanin synthesis to protect the skin against ultraviolet radiation and other external stresses. The loss of skin pigmentation is closely related to depigmented skin disorders. The melanogenic effects of pinostrobin, an active flavanone found in honey, were evaluated. B16F10 cells were used for melanin content, tyrosinase activity, and the expression of melanogenesis-related markers. Moreover, computational simulations were performed to predict docking and pharmacokinetics. Pinostrobin increased melanin levels and tyrosinase activity by stimulating the expression of melanogenic regulatory factors including tyrosinase, tyrosinase-related protein (TRP) 1 and microphthalmia transcription factor (MITF). Specifically, the phosphorylation of cAMP response element binding (CREB) involved in the MITF activation was augmented by pinostrobin. Moreover, the compound upregulated the β-catenin by cAMP/PKA-mediated GSK-3β inactivation. Co-treatment with a PKA inhibitor, inhibited melanin production, tyrosinase activity, and expression of MITF, *p*-CREB, *p*-GSK-3β and *p*-β-catenin, demonstrating that pinostrobin-stimulated melanogenesis was closely related to cAMP/PKA signaling pathway. Furthermore, the combination of pinostrobin and a specific p38 inhibitor, showed that MITF upregulation by pinostrobin was partly associated with the p38 signaling pathway. Docking simulation exhibited that the oxygen group at C-4 and the hydroxyl group at C-5 of pinostrobin may play an essential role in melanogenesis. In silico analysis revealed that pinostrobin had the optimal pharmacokinetic profiles including gastrointestinal absorption, skin permeability, and inhibition of cytochrome (CYP) enzymes. From the present results, it might be suggested that pinostrobin could be useful as a potent and safe melanogenic agent in the depigmentation disorder, vitiligo.

## 1. Introduction

Human skin plays a major role in immunologic responses providing a physical barrier that may affect the physiological status of the body. Moreover, the skin acts as an immune system and, through its pigments, provides a protective mechanism against ultraviolet radiation (UVR) [1]. Melanin is responsible for the hair, eyes and skin, pigmentation, which is produced by melanosomes in melanocytes. Melanocytes transfer melanin via their dendrites to adjacent keratinocytes, where they form the melanin caps that reduce UV-stimulated epidermal DNA damage [2]. The loss of skin pigmentation can cause compromised cutaneous immunity, resulting in conditions, such as vitiligo, an acquired skin pigmentation disorder. The main cause of the depigmentation disease is the destruction of melanocytes and the impediment of melanogenesis pathways [3].

Melanogenesis, a biosynthetic pathway for melanin production, is mediated by three specific enzymes, tyrosinase, tyrosinase related protein 1 (TRP 1) and TRP 2 [4]. Tyrosinase, the rate-limiting enzyme in melanin synthesis, catalysis the hydroxylation of tyrosine into dihydroxyphenylalanine (DOPA) and the further oxidation of DOPA into DOPA quinone. Aside from tyrosinase, TRP 1 and TRP 2 reside in melanosomes and also regulate melanin production [5]. In mammals, two types of melanin are produced, pheomelanin (yellow/red) and eumelanin (brown/black). Tyrosinase is a common enzyme required for both types of melanin synthesis, whereas TRP 1 and TRP 2 are more specific for eumelanin formation. These tyrosinase family genes are controlled by microphthalmia transcription factor (MITF), a master regulator of melanocyte proliferation and survival [6].

When exposed to UVR, α-melanocyte stimulating hormone (α-MSH) is released and binds to melanin cortin-1 receptor (MC1R), resulting in the activation of cyclic adenosine monophosphate (cAMP) [7]. cAMP subsequently stimulates cAMP-dependent protein kinase A (PKA) and the catalytic subunit of PKA translocates to the nucleus where it phosphorylates cAMP-response element binding (CREB). The phosphorylated active CREB further binds MITF, which in turn stimulates the transcription of the key melanogenic enzymes [8].

β-Catenin signaling is one of the pathways involved in MITF expression, which contributes to melanogenesis. In normal conditions, the level of cytoplasmic β-catenin is kept low via multiprotein complexes, such as Axin, APC, GSK-3 and CK1 mediated degradation [9]. Previous studies demonstrated that activating the cAMP/PKA pathway stimulates the phosphorylation of GSK-3β at Ser 9, facilitating β-catenin accumulation [10,11]. The increased β-catenin is transported into the nucleus in the form of a complex with the lymphoid-enhancing factor-1/T cell factor (LEF-TCF) transcription factor, and then promotes MITF expression [12].

Mitogen-activated protein kinases (MAPKs) including extracellular responsive kinase (ERK), c-Jun *N*-terminal kinase (JNK), and p38 MAPK are major signaling molecules associated with the regulation of melanogenesis [13]. The phosphorylated p38 activates MITF to ultimately stimulate melanin synthesis, while the activation of ERK 1/2 and JNK leads to a decrease in melanogenesis via MITF degradation [14].

Natural products with diverse structures are well recognized as important sources in the development of safe and effective therapeutic agents for diseases including cancers, inflammation, dementia, vitiligo, etc. [15]. In our ongoing research of exploring natural melanogenic activators, 50 well-known natural compounds from foods were screened for stimulating effects on tyrosinase activity and cellular melanin production. Among them, pinostrobin, a major flavonoid found in honey, propolis, and finger roots, had the highest activity in producing melanin compared with the other flavonoids, such as quercetin, catechin, resveratrol, hesperidin, naringenin, etc. In the present study, the melanogenic activity of pinostrobin and its possible underlying cAMP/PKA and MAPK signaling pathways were elucidated for the first time.

## 2. Materials and Methods

### 2.1. Chemicals and Reagents

Pinostrobin (5-hydroxy-7-methoxyflavanone) (≥99%), MTT reagent, H89, PD98059, SB203580, and L-DOPA were purchased from Sigma-Aldrich (St. Louis, MO, USA). *p*-CREB (Ser133), *p*-GSK-3β (Ser9), *p*-β-catenin (Ser675), *p*-ERK, *p*-JNK, *p*-p38, and p38 antibodies were purchased from Cell Signaling Technology Inc. (Beverly, MA, USA), whereas antibodies for tyrosinase, TRP 1, TRP 2, MITF, GSK-3β, JNK, ERK, β-actin, mouse IgG-HRP, and rabbit antibody IgG-HRP were obtained from Santa Cruz Biotechnology Inc. (Santa Cruz, CA, USA).

### 2.2. Cell Lines and Pinostrobin Treatment

B16F10 cell was purchased from the Korean Cell Line Bank (Seoul, Korea) and cultured in DMEM supplemented with 10% FBS and 1% penicillin/streptomycin (all from Hyclone, Logan, UT, USA) at 37 °C in a humid atmosphere of 5% CO_2_. A stock solution of pinostrobin was prepared by dissolving DMSO. Control cells were treated with equal volumes of DMSO.

### 2.3. Cell Viability Assay

The cells (3 × 10^3^ cells/well) were seeded into 96-well and cultured for 24 h. The medium was then replaced with fresh medium containing pinostrobin at various concentrations (25 µM, 50 µM, 100 µM, or 200 µM) and incubated for 3 days. Next, the cells were treated with MTT solution (5 mg/mL) at 37 °C for 3 h. Then, the MTT solution was removed and DMSO was added to dissolve. Absorbance was measured at 570 nm using a microplate spectrophotometer (ELX808, BioTek, Winooski, VT, USA).

### 2.4. Measurement of Melanin Content

Melanocytes were exposed to pinostrobin for 3 days of culture and washed. The cells were excised with trypsin-EDTA and the culture medium was removed by centrifugation. The pellet was then incubated in 1N NaOH solution at 80 °C for 2 h to dissolve melanin. The melanin contents were measured by an ELISA reader (405 nm) and presented as a percentage of those of untreated controls [16].

### 2.5. Evaluation of Tyrosinase Activity

The cells (2.0 × 10^5^) were seeded in 6-well plates and stimulated with pinostrobin for 3 days. The extracted protein content was assessed using a BCA Protein Assay kit (Thermo Scientific Pierce, Rockford, IL, USA). L-DOPA (10 mM) was added to each well of a 96-well plate. Then, potassium phosphate buffer (0.1 M, pH 6.8) and cell protein lysate (1 µg/µL) were added. The reaction mix was incubated for 1 h at 37 °C and then measured at 490 nm [17].

### 2.6. Western Blot Analysis

After pinostrobin treatment, the cells were harvested and lysed. The cell lysates (25 µg) were mixed with 5X Laemmli buffer (Elpis Biotech, Daejeon, Korea) and boiled for 5 min. The samples were divided using sodium dodecyl sulfate-polyacrylamide gels and transferred to a polyvinylidene difluoride membrane (Amersham Biosciences, Piscataway, NJ, USA). The membranes were blocked in 5% skim milk and incubated for 24 h at 4 °C with diluted primary antibodies: β-actin (1:10,000), MITF (1:500), tyrosinase (1:500), TRP 1 (1:2000), TRP 2 (1:1000), *p*-CREB (1:1000), *p*-GSK-3β (1:2000), GSK-3β (1:1000), *p*-β-catenin (1:1000) *p*-ERK (1:3000), ERK (1:3000), *p*-p38 (1:2000), p38 (1:2000), *p*-JNK (1:1000), and JNK (1:1000). Then, the membranes were then further incubated with a secondary antibody corresponding to the primary antibody. The protein band was detected using an ECL kit (Advansta Inc, Menlo Park, CA, USA) and photographed with the Atto EZ-capture system (Tokyo, Japan). The intensities of the protein bands were performed using the ImageJ software (NIH, Bethesda, MD, USA). Each protein detection had three replicates.

### 2.7. In Silico Pharmacokinetic Prediction and Docking Simulation

SwissADME (http://www.swissadme.ch, accessed on 19 July 2022 Swiss Institute of Bioinformatics, Lausanne, Switzerland) is a web-based tool to generate physicochemical descriptors, such as molecular weight, molecular refractivity, count of specific atom types, polar surface area, lipophilicity and water solubility. It also calculates pharmacokinetic properties including human gastrointestinal absorption, caco-2 permeability, skin permeability coefficient and interaction of molecules with cytochromes p450 (CYP). The SMILES (Simplified Molecular Input Line Entry System) format of pinostrobin generated from ChemSketch software 2012 version (ACD/Labs., Toronto, ON, Canada) was entered in the SwissADME [18].

The 3D structure of pinostrobin (73201) was obtained from PubChem. The crystal structure of PKA (ID No. 1CX4), and p38 (ID No. 3ZS5) was retrieved from the Protein Data Bank (PDB). Docking simulation was performed using AutoDock Vina 1.1.2 (The Scripps Research Institute, La Jolla, CA, USA) [19]. The best docking mode was the highest ligand-binding affinity. RMSD values of the lower bound and the upper bound were zero. The dimensions of the box were set to 40 Å × 40 Å × 40 Å, which is large enough to cover the entire binding site, and the default exhaustiveness value was 4. Poses were visually assessed and analyzed through PyMOL 2.5.0 version (Schrödinger, Inc., New York, NY, USA). The pharmacophore of hydrogen bonds and Van der Waals interactions were conducted using the Ligplot+ program version 2.2 (EMBL-EBI, Hinxton, Cambridgeshire, UK).

### 2.8. Statistical Analysis 

Statistical analysis was conducted with ANOVA followed by Tukey’s multiple comparisons test. * *p* < 0.05, ** *p* < 0.01, and *** *p* < 0.001 were considered statistically significant differences.

## 3. Results

### 3.1. Effects of Pinostrobin on Melanin Production and Tyrosinase Activity in B16F10 Cells

**Figure 1:**
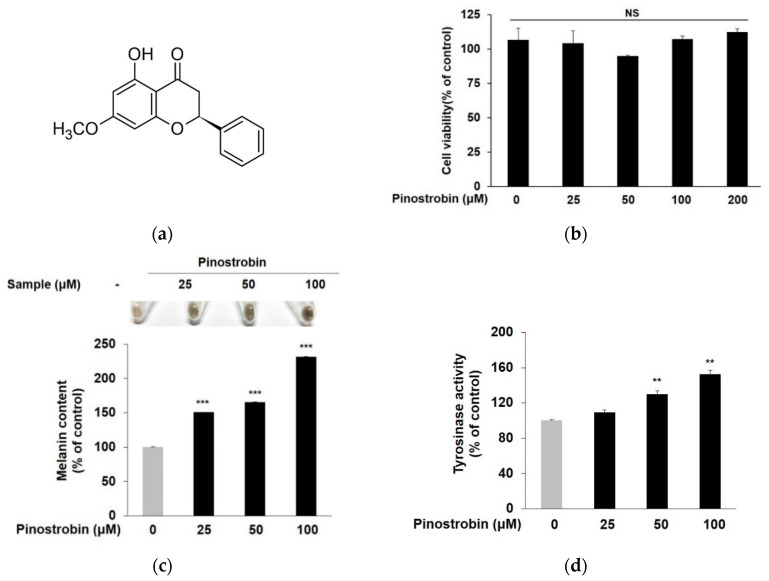
Effect of pinostrobin on cell viability, intracellular melanin contents, tyrosinase activity in B16F10 cells. (**a**) Chemical structure of pinostrobin. (**b**) After treatment with various concentrations of pinostrobin for 72 h, the MTT assay was performed to analyze the cell viability. (**c**,**d**) After treatment under the same conditions used for the determination of cell viability, cells were collected and lysed to measure intracellular melanin content and tyrosinase activity. Cell viability, intracellular melanin contents, and tyrosinase activity in control cells were regarded as 100%. *** *p* < 0.001 and ** *p* < 0.01 compared with control group. NS; not significant.

The structure of pinostrobin purchased from Sigma-Aldrich was revealed in Figure 1a. As shown in Figure 1b, it did not exhibit any cytotoxicity in the range from 25 to 200 μM in B16F10 cells. For further experiments, doses below 100 μM were selected. The compound significantly increased the melanin content at all tested concentrations (Figure 1c). In particular, the melanin production was noticeably elevated more than 2.3 times after 100 μM of pinostrobin pretreatment.

Since tyrosinase is the rate-limiting enzyme for regulating melanin production, increasing the activity of tyrosinase has become a target for the development of melanogenic agents. As shown in Figure 1d, pinostrobin exhibited a somewhat weaker effect at 25 μM, but considerably augmented intracellular tyrosinase activity at 50 μM and 100 μM (130.1 ± 3.9% and 152.7 ± 4.5%, respectively).

### 3.2. Pinostrobin Regulated the Protein Expression of Melanogenic Enzymes and MITF

**Figure 2:**
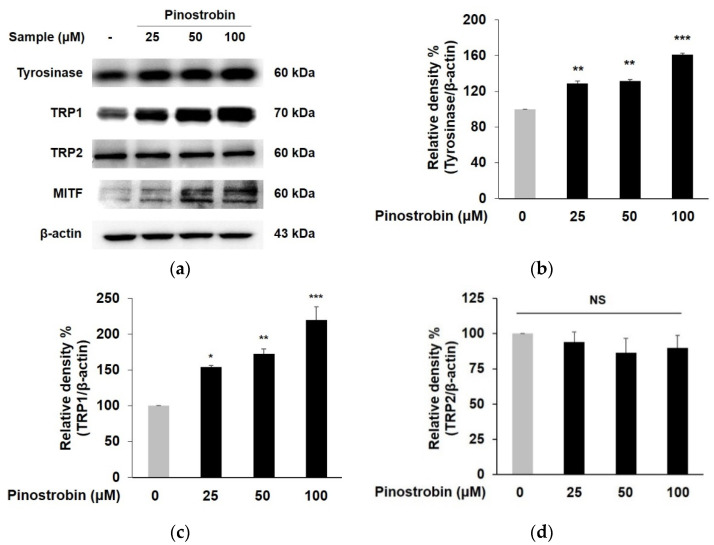

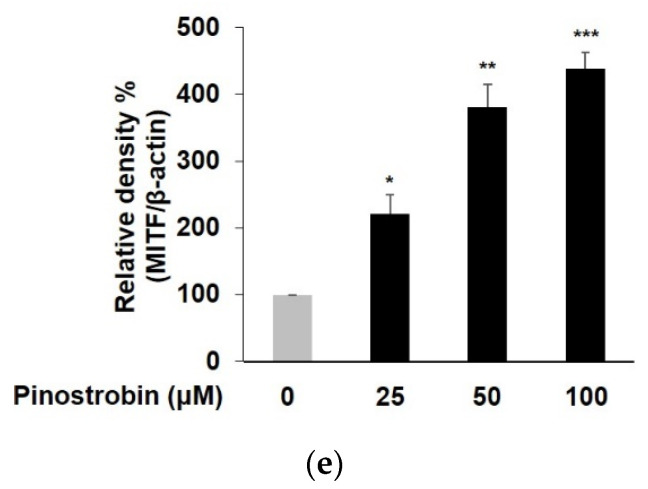
Effect of pinostrobin on the protein levels of tyrosinase, TRP 1, TRP 2, and MITF in B16F10 cells. Cells were treated with pinostrobin at various concentrations for 24 h. (**a**) Tyrosinase, TRP 1, TRP 2, and MITF, were analyzed by Western blotting. (**b**–**e**) Blots were quantified using ImageJ software. β-actin is used as a loading control for quantitative Western blotting. The untreated cells were regarded as 100%. Values are expressed represent as the mean±SD of three independent experiments. *** *p* < 0.001, ** *p* < 0.01 and * *p* < 0.05 compared with control group. NS; not significant.

To determine whether stimulation of tyrosinase activity by pinostrobin was caused by the regulation of the tyrosinase gene family, Western blotting was performed. As shown in Figure 2a–c, the expression of tyrosinase and TRP 1 was significantly augmented by the pretreatment with pinostrobin at all concentrations, in particular, it increased to 1.6-fold and 2.1-fold, respectively, with the highest concentration of pinostrobin. On the contrary, pinostrobin showed no effects on TRP 2 expression (Figure 2d).

Since MITF plays a crucial role in melanogenesis as a key transcriptional modulator of tyrosinase and TRPs, MITF expression was further investigated [6]. As depicted in Figure 2a,e, the tested compound significantly induced MITF expression to 2.2-fold, 3.8-fold, and 4.4-fold at a concentration of 25–100 μM, respectively, which clearly demonstrated that the melanogenic effect of pinostrobin was promoted by MITF-dependent activation of tyrosinase and TRP 1.

### 3.3. Pinostrobin Mediated Melanogenesis by Upregulating CREB, GSK-3β and β-Catenin

CREB, GSK-3β, and β-catenin are transcription factors that are closely related to MITF-dependent melanogenesis [20]. CREB activates MITF transcription, resulting in increasing melanin synthesis. In addition, phosphorylated GSK-3β induces β-catenin translocation, where it upregulates MITF and thus promotes melanogenic gene expression [9].

**Figure 3:**
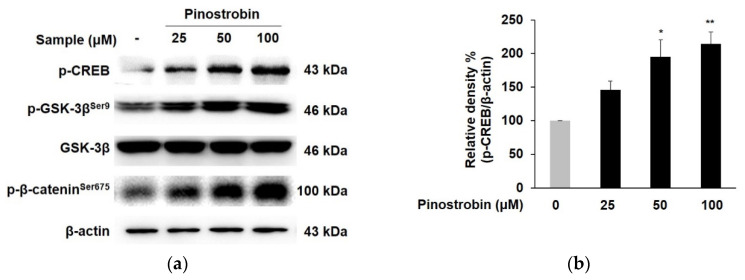

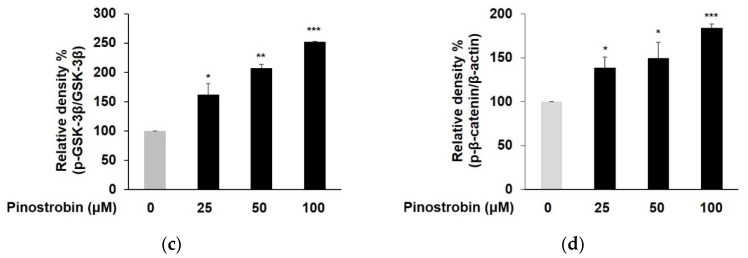
Effect of pinostrobin on the phosphorylation of CREB, GSK-3β, and β-catenin in B16F10 cells. Cells were treated with pinostrobin at various concentrations for 24 h. (**a**) *p*-CREB, *p*-GSK-3β, and β-catenin, were analyzed by Western blotting. (**b**–**d**) Blots were quantified using ImageJ software. Protein levels were normalized to the corresponding loading controls. The untreated cells were regarded as 100%. Values are expressed represent as the mean ± SD of three independent experiments. *** *p* < 0.001 ** *p* < 0.01, and * *p* < 0.05 compared with control group.

To define the mechanisms underlying the melanin synthetic property of pinostrobin, changes in the upstream activator of MITF induced by pinostrobin were examined. As depicted in Figure 3a,b, pinostrobin at 50 and 100 μM promoted the phosphorylation of CREB more than two-fold compared to the control group. The compound obviously elevated GSK-3β phosphorylation without significantly affecting total GSK-3β expression (Figure 3a,c). Particularly, the expression level of phosphorylated GSK-3β was dramatically elevated (>2 folds) after pretreatment with pinostrobin at 50 and 100 μM. Consistent with the results of GSK-3β, the level of phosphorylated β-catenin was significantly augmented in pinostrobin-treated cells (Figure 3a,d).

### 3.4. Pinostrobin Controlled the Melanogenesis via PKA Dependent Signaling Pathway

**Figure 4:**
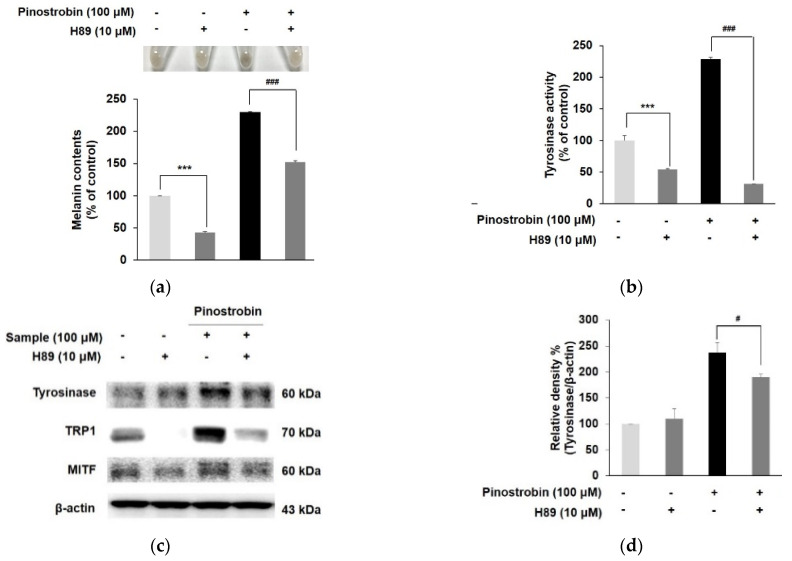

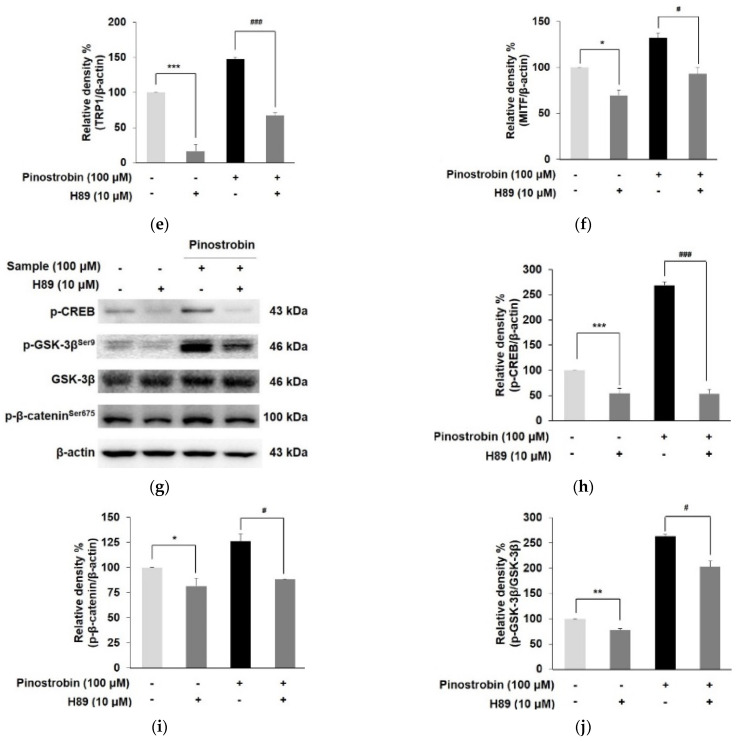
Effect of pinostrobin and H89 (PKA inhibitor) on PKA signaling pathway in B16F10 cells. Cells were pre−treated with H89 for 30 min prior to exposure to pinostrobin (100 μM) for 72 h to evaluate (**a**) the intracellular melanin contents, and (**b**) tyrosinase activity. (**c**–**f**) Cells were treated with H89 (10 μM) for 30 min, followed by treatment with pinostrobin (100 μM) for 24 h. Subsequently, tyrosinase, TRP 1, and MITF were analyzed by Western blotting. (**g**–**j**) Western blotting result of *p*-CREB, *p*-GSK-3β, and *p*-β-catenin expression with co-treatment of H89 and pinostrobin. Blots were quantified using ImageJ software. Protein levels were normalized to the corresponding loading controls. The untreated cells were regarded as 100%. Values are expressed represent as the mean±SD of three independent experiments. *** *p* < 0.001, ** *p* < 0.01, and * *p* < 0.05 compared with control group; ### *p* < 0.001 and # *p* < 0.05 compared with pinostrobin group without H89.

To verify the involvement of the cAMP/PKA cascade in pinostrobin-mediated melanogenesis, the specific PKA inhibitor (H89) was used. As shown in Figure 4a,b, H89 at 10 µM markedly reduced the level of melanin contents and tyrosinase activity compared with the H89 untreated control. Co-treatment of pinostrobin and H89 significantly reduced the pinostrobin-induced melanin synthesis and tyrosinase activity (*p* < 0.001). In addition, pinostrobin in combination with H89 obviously decreased the expression of tyrosinase, TRP 1, and MITF more than only pinostrobin did, as illustrated in Figure 4c–f. Moreover, upregulated phosphorylation of CREB, GSK-3β, and β-catenin induced by pinostrobin at 100 μM was markedly attenuated when co-treated with H89 (Figure 4g–j), implying that the cAMP/PKA signaling pathway is directly related to pinostrobin-mediated melanogenesis.

### 3.5. Pinostrobin Regulated p38 and ERK Signaling Pathway

**Figure 5:**
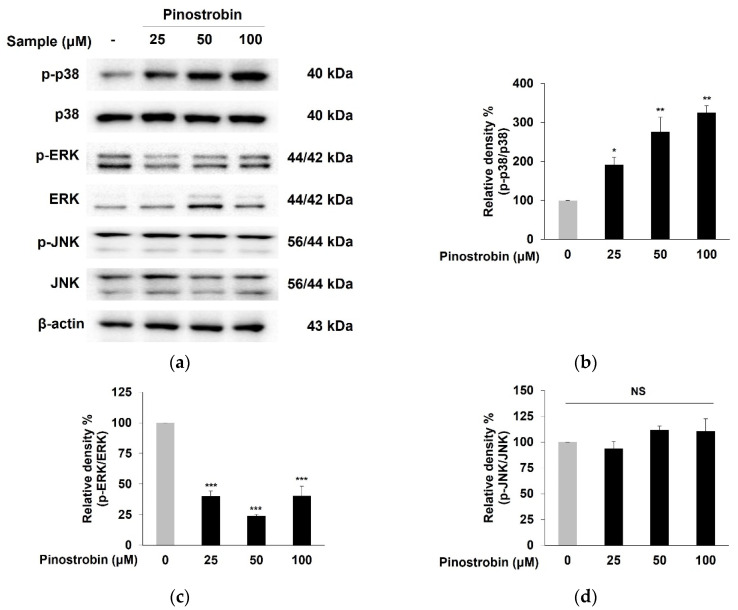
Effect of pinostrobin on the phosphorylation of p38, ERK, and JNK in B16F10 cells. (**a**–**d**) Cells were treated with pinostrobin at various concentrations for 24 h and then harvested. *p*-p38, *p*-ERK, and *p*-JNK, were analyzed by Western blotting. Blots were quantified using ImageJ software. Protein levels were normalized to the corresponding loading controls. The untreated cells were regarded as 100%. Values are expressed represent as the mean ± SD of three independent experiments. *** *p* < 0.001 ** *p* < 0.01, and * *p* < 0.05 compared with control group. NS; not significant.

MAPKs were demonstrated as one of the upstream pathways of melanogenesis by regulating MITF activation [14]. The phosphorylation of p38, ERK, and JNK was evaluated to elucidate the mechanism underlying the MITF-mediated melanogenesis property of pinostrobin. As shown in Figure 5a–c, the levels of p38 phosphorylation were remarkably elevated, while the ERK phosphorylation was significantly reduced in pinostrobin-treated cells as compared to those of the control. However, the compound had no effect on phosphorylated JNK (Figure 5a,d). These results indicated that both p38 activation and ERK suppression were partly related to melanogenesis by pinostrobin.

### 3.6. Pinostrobin Stimulated Melanogenesis via p38 Signaling Pathway

**Figure 6:**
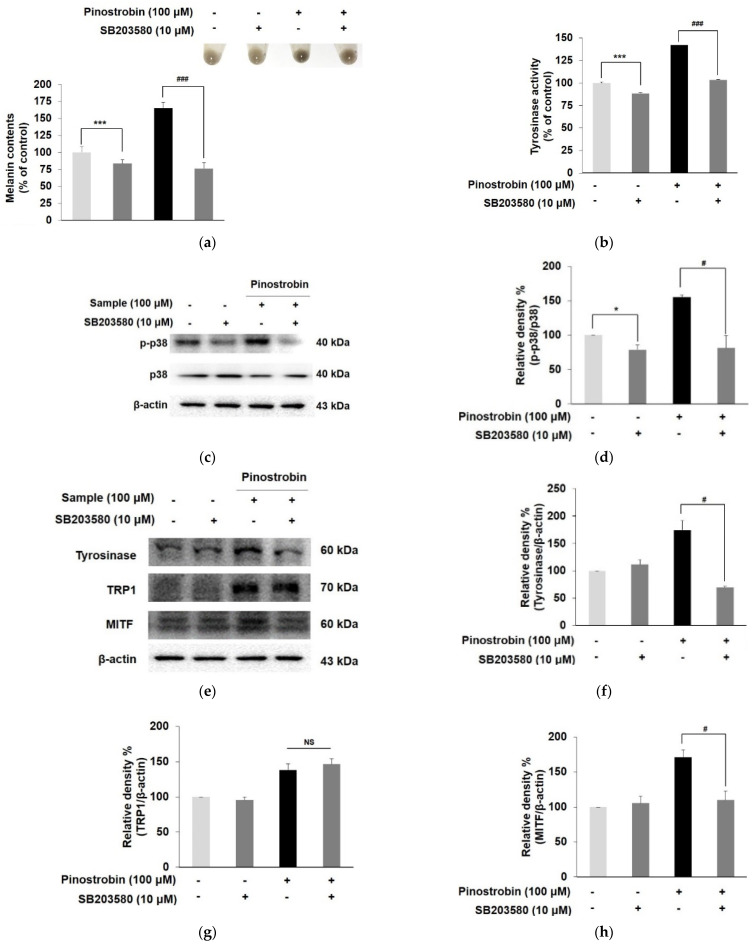

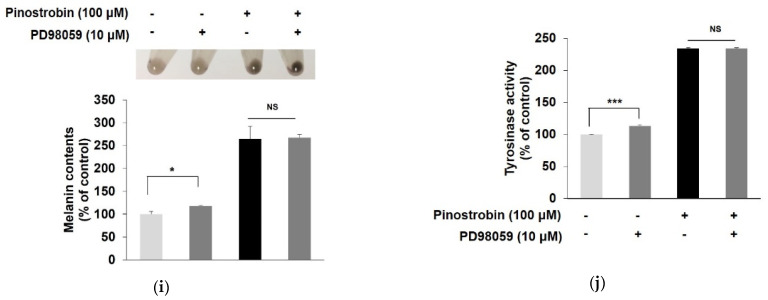
Effect of pinostrobin with SB203580 (p38 inhibitor) or PD98059 (ERK inhibitor) on MAPK signaling pathway in B16F10 cells. Cells were pre-treated with SB203580 (10 μM) and pinostrobin (100 μM) for 72 h to evaluate (**a**) the intracellular melanin contents, and (**b**) tyrosinase activity. B16F10 cells were treated with SB203580 (10 μM) for 30 min, followed by treatment with pinostrobin (100 μM) for 24 h. Subsequently, the expression of (**c**,**d**) *p*-p38, (**e**,**f**) tyrosinase, (**g**) TRP 1 and (**h**) MITF was analyzed by Western blotting. B16F10 cells were co-treated with PD98059 (10 μM) and pinostrobin (100 μM) for 72 h to evaluate (**i**) the intracellular melanin contents, and (**j**) tyrosinase activity. Blots were quantified using ImageJ software. Protein levels were normalized to the corresponding loading controls. The untreated cells were regarded as 100%. Values are expressed represent as the mean ± SD of three independent experiments. *** *p* < 0.001, and * *p* < 0.05 compared with control group; ### *p* < 0.001 and # *p* < 0.05 compared with pinostrobin group without SB203580 or PD98059. NS; not significant.

To confirm the involvement of p38 and ERK phosphorylation in the melanogenic effect of pinostrobin, the expression of melanogenic-related markers was evaluated in the presence of pinostrobin and MAPK inhibitors. When the p38 signaling pathway was blocked by co-treating the cells with a specific p38 inhibitor SB203580 and pinostrobin, the melanin contents and tyrosinase activity considerably decreased compared to cells treated with pinostrobin alone (Figure 6a,b). Moreover, the combination of pinostrobin and SB203580 noticeably reduced the expression of MITF and its downstream targets tyrosinase as well as the p38 expression induced by pinostrobin, suggesting that the compound increased tyrosinase activity and expression via p38-mediated upregulation of MITF (Figure 6c–h). As illustrated in Figure 6i,j, ERK inhibitor (PD98059) significantly induced melanin production and tyrosinase activation. However, the co-treatment of pinostrobin and PD98059 showed no effect on melanin contents and tyrosinase activity, indicating the contributions of ERK signaling are relatively weaker than p38 in the MAPK pathway. These results demonstrated that the p38 pathway was closely associated with the MITF-mediated melanogenesis by pinostrobin.

**Figure 7:**
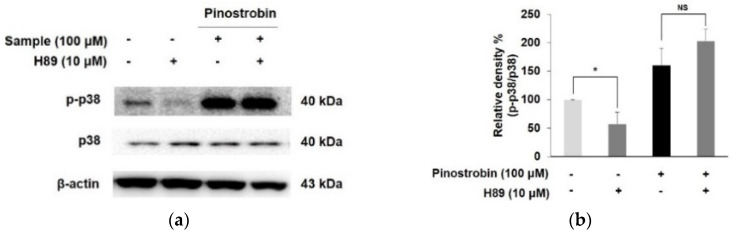
Effect of pinostrobin with H89 on phosphorylated p38 expression in B16F10 cells. (**a**) Cells were pre−treated with H89 for 30 min prior to exposure pinostrobin (100 μM) for 24 h and then harvested. (**b**) Blots were quantified using ImageJ software. Protein levels were normalized to the corresponding loading controls. The untreated cells were regarded as 100%. Values are expressed represent as the mean ± SD of three independent experiments. * *p* < 0.05 compared with control group. NS; not significant.

To validate the contribution of the PKA signaling pathway to pinostrobin-induced p38 phosphorylation, B16F10 cells were co-treated with PKA inhibitor and pinostrobin (Figure 7a,b). H89 alone decreased the phosphorylation level of p38 MAPK, whereas co-treatment with pinostrobin and H89 did not change the expression of *p*-p38, suggesting that pinostrobin-induced PKA and p38 signaling pathways were independent.

### 3.7. Molecular Docking Simulation of Pinostrobin

**Figure 8:**
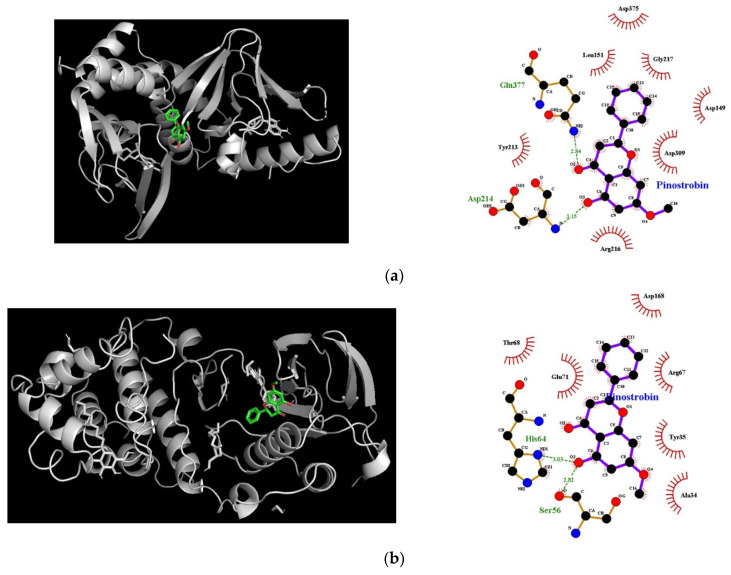
Molecular docking interactions of pinostrobin with (**a**) PKA and (**b**) p38; surface view, and interaction map, such as hydrogen (dotted line in green) and hydrophobic bonding (red dashed semicircle) between pinostrobin with upstream targets involved in melanogenesis.

Table 1 and Figure 8 showed the molecular docking results of pinostrobin with biomarkers in the cAMP/PKA and p38 MAPK signaling pathway. Two hydrogen bonds were found between pinostrobin and PKA residues with the lowest binding energy of –7.3 kcal/mol. The Asp214 and Gln377 residues in PKA participated in hydrogen interactions with the hydroxyl group at C-5 and oxygen group at C-4 of pinostrobin (bonding distances of 2.84 Å and 3.15 Å, respectively). The binding site for the PKA-pinostrobin complex was formed by van Der Waals interactions with Asp149, Leu151, Tyr213, Arg216, Gly217, Asp309, and Asp375 (Table 1 and Figure 8a).

In the p38-pinostrobin complex, the lowest binding energy between pinostrobin and p38 was −7.6 kcal/mol (Table 1 and Figure 8b). Six residues of p38, such as Ala34, Tyr35, Arg67, Thr68, Glu71, and Asp168 were demonstrated to participate in van Der Waals interactions with the compound. Interestingly, the hydroxyl group at C-5 of pinostrobin simultaneously bound at Ser56, and His64 residues of p38 with bonding distances of 2.82 Å and 3.03 Å, respectively.

### 3.8. In Silico SwissADME Profile of Pinostrobin

Pharmacokinetic parameters are needed to understand the efficacy and safety of the target compound in vivo. Designing novel candidates requires substantial attention to their pharmacokinetic properties including intestinal absorption, penetration of the skin, and inhibition of CYP isoforms.

As shown in Table 2, pinostrobin was predicted to be absorbed in the gastrointestinal (GI) tract and penetrated the stratum corneum, the rate-limiting barrier for skin penetration. In addition, the compound was predicted as an inhibitor of several CYP enzymes including CYP1A2 and CYP2C19.

## 4. Discussion

Vitiligo is the most common disease of acquired depigmentation adversely affecting patient quality of life [21]. It has been reported that around 1% of the world population has vitiligo, regardless of skin type or gender [22]. The etiological factor of vitiligo is still obscure and several different hypotheses have been suggested, which involve a combination of susceptibility genes and environmental factors contributing to the autoimmune destruction of melanocytes [23]. To date, the therapeutic options for vitiligo are limited and are mostly based on the use of immunosuppressive agents including topical corticosteroids, and UV light treatment. However, those treatments not only lack sustained efficacy, but also have side effects, such as scales, skin atrophy, pustules, telangiectasia, and local endosymbiosis proliferation [24]. Recently, Janus kinase (JAK)–signal transducer and activator of transcription (STAT) have been shown to be involved in the pathogenesis of vitiligo [25]. Coumarin-neurotransmitter derivatives have been reported as promising inhibitors of JAK–STAT signaling. [26]. Moreover, clinical trials have shown that JAK inhibitors including tofacitinib, ruxolitinib, and baricitinibare recovered the pigment loss via alleviating the response of autoimmune and inflammation [25].

Safe and natural compounds that affect melanogenesis may be considered potential preventive and/or therapeutic agents for depigmentation skin disorders [27]. Pinostrobin is the bioactive flavanone found in honey, finger root ginger, etc. [28,29]. Pinostrobin is a chiral flavonoid with two enantiomers. However, in most cases, chiral compounds are produced in nature in optically pure forms, where only one enantiomer is biosynthesized in the producing organism [30,31]. For pinostrobin, only the (-)-isomer of pinostrobin is produced by natural sources, such as honey, propolis, and finger roots [32], where the synthesis of pinostrobin often results in a racemic mixture (50:50) of the two enantiomers. The compound has been reported to have various biological activities, such as antioxidant, anti-inflammatory, anti-leukemia, anti-tumor, and neuroprotective properties [33,34,35].

A novel activity of pinostrobin, the melanogenic property, was demonstrated in the present study. Melanogenesis is a complex process that is related to more than 120 genes [36]. Among the genes, the tyrosinase family enzymes including tyrosinase, TRP 1, and TRP 2 have been recognized as the key regulators of melanogenesis [37]. Melanin is biosynthesized in the melanosome by the action of the tyrosinase and TRPs, which are transcriptionally regulated by MITF. Pinostrobin elevated melanin synthesis and tyrosinase activity by promoting the expression of melanogenic regulatory factors including tyrosinase, TRP 1, and MITF.

The cAMP/PKA phosphorylates the CREB, which is known to be an activator of MITF expression [11]. The activation of the cAMP/PKA pathway also stimulated β-catenin activation through GSK-3β inactivation, leading to increased MITF expression [38]. As a result, the compound enhanced melanogenesis via activating cAMP/PKA pathways and its downstream targets including CREB, GSK-3β, and β-catenin, thus upregulation of MITF protein levels subsequent activation of tyrosinase and TRP 1 enzymes. However, pre-treatment with pinostrobin in the presence of a PKA inhibitor considerably decreased the melanogenic effect of pinostrobin, demonstrating that MITF upregulation is mediated by the cAMP/PKA signaling pathway.

Previous studies have demonstrated that melanogenesis is mediated by the regulation of MITF activation via phosphorylation of p38 MAPK [13,39]. In the present study, pinostrobin augmented the phosphorylation level of p38, whereas the combination of the compound and SB203580 reduced the increasing effect of pinostrobin on the melanin synthesis, tyrosinase activity, expression of MITF and its downstream target tyrosinase, proving that p38 is strongly associated with the melanogenesis effect of pinostrobin.

Compared with the extensive research on the beneficial effects of flavonoids including antioxidant, anti-cancer, and anti-inflammatory activities, relatively little study has been done on their melanogenic effects. Several flavonoids have been found to be involved in melanogenesis [40,41,42,43,44]. Cirsimaritin found in rosemary, exerted a profound effect on melanin synthesis by a similar mechanism as shown with pinostrobin [40]. Major citrus flavonoids, such as hesperetin and naringenin had stimulatory effects on melanin production [41,42,43]. However, hesperidin suppressed melanin synthesis in normal human melanocytes as well as in B16F10 cells despite its structural similarity [44]. Considering the structures of the above melanogenic compounds, it can be suggested that the C-5 hydroxyl group and C-7 methoxy group may partially play an important role in melanogenic activity.

Molecular docking simulation is a well-established and widely used method in the drug discovery process, which predicts ligand–target interactions at the molecular level, allowing the identification of novel compounds of therapeutic interest. This approach also explores binding affinity and structure-relationship by evaluating critical phenomena involved in the intermolecular recognition process. In fact, the results of the in silico docking simulation revealed that the oxygen group at C-4 and the hydroxyl group at C-5 of pinostrobin were responsible for molecular interaction, suggesting these functional groups were determinants of its biological function. Moreover, it revealed that pinostrobin entrenched specific interactions with the key amino acid residues of melanogenic related markers via hydrogen bonding and van der Waals interactions, demonstrating the compound has an appropriate molecular structure to promote melanin synthesis.

Pharmacokinetic profiling, such as absorption, distribution, metabolism, and excretion (ADME) processes, is important to understanding the in vivo behavior and mechanism of action of a candidate. To date, there have been several studies regarding the pharmacokinetic properties of pinostrobin in rats [45,46,47]. Pinostrobin was mainly distributed in the gastrointestinal tract in rats after a single oral administration [45]. The compound in the parent form exhibited less than 1.6% elimination into the urine, bile and feces, suggesting that pinostrobin was mostly metabolized in vivo and played a role in different organs [45,46]. In addition, pinostrobin at 1–100 mg/kg for 7 days showed no mutagenic effect in Wister rats, further confirming the safety consumption of the compound [47]. Although there has been no experimental study on the skin permeation of pinostrobin so far, our computational prediction of the skin permeability coefficient (Log Kp) by the octanol–water partition coefficient and molecular size exhibited that pinostrobin can penetrate the skin [48]. The CYPs are a superfamily of heme-containing isoenzymes located mainly in the intestine and liver which play a vital role in drug metabolism [49]. Evaluating the potential of a compound to inhibit a CYPs is important, as the co-administration of compounds may result in one or both inhibiting the other’s metabolism. In the present study using in silico prediction, pinostrobin was found to be an inhibitor of CYP1A2 and CYP2C19. It should be avoided as an alternative supplement during the administration of drugs (substrate of CYP1A2 and CYP2C19) as it is likely to cause food–drug interactions. These results may help to understand CYP-mediated food–drug interactions and evaluate the safety profile of natural products used therapeutically.

## 5. Conclusions

The present study revealed, for the first time, that pinostrobin exerted a stimulatory effect on tyrosinase activity and melanin production without cytotoxicity by inducing expression of tyrosinase, TRP 1 and MITF. The compound increased phosphorylation of CREB and GSK-3β, and subsequently promoted activation of β-catenin, suggesting that pinostrobin induced melanogenesis via regulating the cAMP/PKA signaling pathway. The upregulation of MITF and its downstream targets by pinostrobin was also associated with the p38 MAPK signaling pathway. Furthermore, molecular docking analysis revealed the pinostrobin possessed an appropriate molecular structure for potent interactions with major melanogenesis proteins. In silico prediction also found that the compound had optimal pharmacokinetic properties, but further study is needed to validate skin permeability in biological evaluation. Therefore, it can be suggested that our compound could be used as a novel, potent and safe melanogenic agent for the prevention and/or treatment of vitiligo.

## Figures and Tables

**Table 1:** Docking simulation of pinostrobin with upstream targets involved in melanogenesis.

Target Protein	Binding Energy (kcal/mol)	No. of H- Bonds	H-Bonding Residues	H-Bond Length (Å)	van Der Waals Residues
PKA	–7.3	2	Asp214, Gln377	3.15 2.84	Asp149, Leu151, Tyr213, Arg216, Gly217, Asp309, Asp375
p38	–7.6	2	Ser56, His64	2.82 3.03	Ala34, Tyr35, Arg67, Thr68, Glu71, Asp168

**Table 2:** Pharmacokinetic properties of pinostrobin.

Properties	Predicted Value	Standard Value
Permeability of Caco-2 (log Papp in 10^−6^ cm/s)	1.3	Caco-2 permeability >0.90 (high)
Human intestinal absorption (%)	93.8	<30% (poorly absorbed)
Skin Permeability (log Kp)	–2.8	Log Kp >–2.5 (low)
CYP3A4 inhibitor	No	
CYP1A2 inhibitor	Yes	
CYP2C19 inhibitor	Yes	
CYP2C9 inhibitor	No	
CYP2D6 inhibitor	No	

## Data Availability

Not applicable.
